# An investigation on parenting stress of children with cystic fibrosis

**DOI:** 10.1186/s13052-020-0795-7

**Published:** 2020-03-18

**Authors:** Grazia Isabella Continisio, Nicola Serra, Assunta Guillari, Maria Teresa Civitella, Angela Sepe, Silvio Simeone, Gianpaolo Gargiulo, Silvia Toscano, Maria Rosaria Esposito, Valeria Raia, Teresa Rea

**Affiliations:** 1 Department of Translational Medical Sciences, University Federico II of Naples, Naples, Italy; 2 Department of Molecular Medicine and Medical Biotechnology, University Federico II of Naples, Naples, Italy; 3 Department of Public Health, University Federico II of Naples, Naples, Italy; 4 Department of Biomedicine and Prevention, University of Rome Tor Vergata, Rome, Italy; 5 Istituto Nazionale Tumori Fondazione “G. Pascale”, Naples, Italy

**Keywords:** Parenting stress index, Cystic fibrosis, Child, Clinical management, Biopsychosocial model

## Abstract

**Background:**

The management of chronic diseases, particularly in children, requires an integrated physical and psychological approach to both sick children and their family. This is the case of Cystic Fibrosis (CF), a complex genetic chronic disease, where, a comprehensive evaluation of the emotional impact and an effective multidimensional approach are indicated.

**Aim:**

This study investigates on parenting stress in children and adolescents with CF and its determinants related to parents, children and the disease severity.

**Methods:**

The study involved 34.04% adult males and 65.96% adult females (range 21-55 years) and 47 children with CF, 54.35% males and 45.65% females (range 1-17 years). The data were obtained through a *Parenting Stress Index – Short Form* (PSI-SF) questionnaire. According to the PSI-SF scoring system, three types of stress were detected: a typical stress pattern (normal), a high stress pattern (increased) and a defensive response, which may be considered as a high stress feature in children which requires monitoring and clinical evaluation.

**Results:**

This study shows a significant presence of stress in females (60.23%), of subject *married* (84.62%), unemployed (69.23%) and with education level such as “*middle School*” (61.54%). Concerning children of parents with high stress, it resulted most frequent children with one sibling (53.85%). Finally, by univariate analysis, it resulted a significant positive correlation between parenting stress and disease degree of children. Instead by multivariate analysis, we found that the variables: *Number of siblings* and *Birth order* were a significant positive and negative predictor of parenting stress respectively.

**Conclusion:**

An increased stress level was detected in less than one third of parents of subjects with CF. These data may be related to the psychological support which is part of the routine management of CF care team. However, as children’s features seem to act as a determinant of stress more than parental ones, the parental-child dysfunction should be the target for further integrated interventions.

## Background

Cystic Fibrosis (CF) is the most common serious genetic disease in Caucasian populations, resulting in clinical symptoms in multiple body systems as lung, sinus, gastrointestinal and reproductive tracts. Standard of care includes monitoring pulmonary function, nutritional status, airway clearance, and infection control [1]. Newer oral agents that are based on specific mutations are providing physicians with the ability to target underlying CFTR gene defects, thus leading to an increased life expectancy over 50 years [2] in patients with CF.

However, there is no evidence that chronic time consuming conventional therapies can be stopped and CF still represents a paradigmatic clinical scenario requiring a multidimensional approach for patients and their families among chronic diseases [3].

With regard to families, it has seemed interesting to investigate the degree of stress in parents of children affected by this disease, as caregivers’ psychological wellness is hardly ever taken into consideration during the treatment paths of pediatric patients. In addition, since parenting stress is a common part of parenting experience and it usually occurs when either parents’ commitment exceeds their own internal resources, we have considered understanding how a parent of a child with CF may feel in the light of both their practical and emotional commitment involved.

There is evidence that shows that caregivers of children with a chronic illness report significantly higher general parenting stress than caregivers of healthy children [4–7]. Furthermore, parenting stress is crucial to understand any family dysfunction and/or psychopathology [8]. However, it still remains unclear whether or not and to what extent parental distress is a major feature of cystic fibrosis [9, 10].

The aim of the study is to understand whether this disease may induce a stress pattern in parents of children and adolescents with CF, analyze parental stress severity as well as identify any further correlations with additional factors.

## Methods

### Sampling and eligibility

This study was performed at the Cystic Fibrosis Unit of the University-Hospital Federico II in Naples. The sample included 47 adult parents (34.04% males and 65.96% females) mean age 38.87, and 47 children (54.35% males and 46.65% females) mean age 8.26.

Informed consent was obtained from all parents of children included in the study. For all participants anonymity was guaranteed. No economic incentives were offered or guaranteed for their participation in the study.

The inclusion criteria required to be a parent of a child affected by cystic fibrosis as well as their informed consent. The Local Ethical Committee approved the study. (Protocol number 2018: 254/2018).

### Instrument

The data were obtained through the *Parenting Stress Index – Short Form* (PSI-SF) questionnaire which was designed by Richard R. Abidin [11, 12]. The PSI-SF is a standardized questionnaire which can be filled in by parents without any specific help and is widely used in the clinical practice. It aims to assess parents distress in relation to their child and the context in which the relationship takes place. The stress evaluated by the questionnaire was defined as the discrepancy perceived by parents between the needs imposed by the circumstances related to parenting and the resources available to address them. The PSI-SF included 36 items which were assessed according to the Likert scale. The answers revealed the extent of agreement or disagreement to the proposed statement. There are five categories of agreement: *strongly agree (SA), agree (A), not sure (NS), disagree (D) and strongly disagree (SD)*. Furthermore, the questionnaire was divided into three parts:

1. Parenting Distress – PD. This part analyses the stress dependent on the parent only and not necessarily on the child. It defines the level of stress that the parent perceives on the basis of his/her parenting ability as well as if there is a possible conflict with the other parent or another family component (Questions 1 to 12).
2. Parent-Child Dysfunctional Interaction - P-CDI. This section examines the parent-child relationship which depends both on the parent and the child. The parent perceives that the relationship with his/her child is different from his/her own expectations and from other parents with their children (Questions 13 to 24).
3. Difficult Child (DC). This area focuses on the parents perception of their child by describing their perception of the difference between their own and other children in relation to their expectations (Questions 25 to 36).

In addition, two further parameters were obtained:

- the Defensive Response (DEF), which studies the respondent’s defensive attitude, i.e. it evaluates the parent’s honesty about the answers.
- Total stress which assesses whether or not the parents stress is globally increased using a global score.

### Procedure to evaluate stress

According to the 5-point Likert scale, each category was associated with a number from 1 to 5 where 5 accounts for “strong agreement” and 1 for “strong disagreement”. In the first step, the defensive responses of items 1, 2, 3, 7, 8, 9 and 11 were added and the resulting figure was recorded in the “Defensive response” box. Subsequently, the scores of the subscales were calculated: items from 1 to 12 were added and the total was entered in the “PD” variable. Then, items from 13 to 24 were added and the total sum was recorded in the “P-CDI” variable. Finally, the total sum of items 25 to 36 was assigned to the “DC” variable. The total stress score was obtained by adding the scores of PD, P-CDI and DC and the result was entered in the “Total Stress” variable.

Depending on child age range, a score or range was identified for each subscale and the relevant percentile corresponding to each score of the PSI-SF Italian version [12] was calculated.

For a better understanding of the severity stress, the results were classified into three categories:

1. from 1^st^ to 10^th^ percentile, the stress pattern was referred to as *suspicious stress*;
2. from 15^th^ to 80^th^ percentile the stress was normal and referred to as *normal stress*;
3. from 85^th^ to 100^th^ percentile the stress was considered as high or clinically significant and it was referred to as *high stress*

### Statistical analysis

The statistical analysis was performed using the Matrix Laboratory (MATLAB) analytical toolbox version 2008 (MathWorks, Natick, MA, USA). The data were expressed in terms of figures and percentages for the categorical variables while the continuous data were determined as the mean ± standard deviation unless otherwise specified. The χ^2^ test and was performed to assess any significant differences in proportions or percentages between the two independent groups. χ^2^ test with Yates continuity correction was used where the χ^2^ test was not appropriate. In addition, the Binomial test was performed to compare two mutually exclusive proportions. Any significant difference between the two means was evaluated by the Student’s t-test. Multiple comparison chi-square tests were carried out to highlight any significant differences among the percentages. In that case, if the chi-square test was significant (*p*-value< 0.05), a residual analysis with post hoc Z-test was performed.

.

Finally, a univariate and multivariate linear regression analysis was performed. The statistical test on Pearson’s linear correlation coefficient (R) was performed with the Student t test, under a null hypothesis of Pearson’s linear correlation coefficient *R* = 0. For the analysis, the dependence variable assumed was *Total stress* which was obtained according to the Parenting stress index (PSI-SF) and *Age of parents*, *Gender of parents*, *Civil status, Job, Education level, Age of children, Gender* of *children, Age at diagnosis, Number of siblings of* CF *child, birth order of* CF *child*. Variables were numerically labelled as follows:

- *Gender*: male = 1 and female = 0
- *Civil status*: married = 1 and not married = 0
- *Job*: worker with income = 1 and unemployed = 0
- *Education*: low school = 1, middle school = 2, high school = 3, higher education degree = 4.
- *Clinical Severity*: low = 1, moderate = 2, severe = 3

All tests with *p*-value (*p*) < 0.05 were considered significant.

## Results

Table 1 shows the characteristics of both parents and children.

**Table 1:** Characteristics of 47 parents and children participating in the study

Parent’s characteristics	Percentage/ mean ± StD	Statistical analysis
*Age*	38.87 y.o. ± 8.01 y.o.	
*Gender*		
Male	34.04% (16/47)	34.04% < 65.96%, *p* < 0.0001 ^a^ (B)
Female	65.96% (31/47)	
*Civil status*		
Not married	10.64% (5/47)	10.64% < 89.36, *p* < 0.0001^a^ (B)
Married	89.36% (42/47)	
*Job*		
Worker with income	48.68% (21/47)	48.68% < 51.32%, *p* = 0.72 (B)
Unemployed	51.32% (26/47)	
*Education level*		*p* < 0.0001 * (C)
Elementary school	2.13% (1/47)	Elementary school ^c^, *p* = 0.0024 (Z)
Middle school	53.19% (25/47)	Middle school ^b^, *p* = 0.0005 (Z)
High school	27.66% (13/47)	
Higher Education	17.02% (8/47)	
Children’s characteristics	Percentage/ mean ± StD	Statistical analysis
*Age*	8.26 y.o. ± 4.83 y.o.	
*Gender*		
Male	54.35% (25/47)	54.35% > 45.65%, *p* = 0.23 (B)
Female	45.65% (22/47)	
*Age at diagnosis*	1.64 y.o ± 1.39 y.o.	
*Number of siblings*		*p* < 0.0001 ^a^ (C)
None	40.43% (19/47)	Zero ^b^, *p* = 0.0344 (Z)
One	42.55% (20/47)	One ^b^, *p* = 0.0161 (Z)
Two	12.77% (6/47)	
Three	4.26% (2/47)	Three ^c^, *p* = 0.0044 (Z)
*Birth order*		*p* < 0.0001 ^a^ (C)
First	59.57% (28/47)	First ^b^, *p* < 0.0001 (Z)
Second	36.17% (17/47)	
Third	2.13% (1/47)	Third ^c^, *p* = 0.0017 (Z)
Fourth	2.13% (1/47)	Fourth ^c^, *p* = 0. 0017 (Z)
*Clinical Severity*		*p* < 0.0001 ^a^ (C)
Low	53.19% (25/47)	Low ^b^, *p* < 0.0141 (Z)
Moderate	34.04%(16/47)	
Severe	10.64%(5/47)	Severe^c^, *p* < 0.0087 (Z)
Not defined	2.13%(1/47)	

+ stress expressed in percentiles and evaluated according the PSI-SF scale, ^a^ = significant test, ^b^ = most frequent, ^c^ = less frequent, B = exact Binomial test, C = Multiple comparison χ^2^ test, Z = Z-test, CY = χ^2^ test with Yates correction, StD = standard deviation

Statistical tests were performed for each variable. As expected, there were more mothers than fathers as key responders (*p* < 0.0001) as well as married versus unmarried parents (89.36% > 10.64%, *p* < 0.0001). More frequently, parents had a “*Middle School*” education level (53.19%, *p* = 0.0005) and 51.32%% reported a regular job.

As to CF children’s features, their mean age was about 8 years and 54.35% of these were males. In addition, children with no (40.43%, *p* = 0.0344) or one (42.55%, *p* = 0.0161) sibling were more frequent whilst the most frequent birth order was “first” child (59.57%, *p* < 0.0001). Finally, in terms of disease severity, the most frequent degree was “low” whereas “severe” was significantly less frequent compared to other severity degrees.

Table 2 reports two groups: Mother and Father.

**Table 2:** Stress evaluation on the total sample, male and female group

Stress evaluation	Male and Female	Male	Female	Male vs Female
PD: Suspicious Stress	4.26% (2/47)	0.00% (0/16)	6.45% (2/31)	0.00% < 6.45%, *p* = 0.78 (CY)
PD: Normal Stress	74.47% (35/47)	87.50% (14/16)	67.74% (21/31)	87.50% > 67.74%, *p* = 0.26 (CY)
PD: High stress	21.28% (10/47)	12.5% (2/16)	25.81% (8/31)	12.5% < 25.81%, *p* = 0.50 (CY)
P-CDI: Suspicious Stress	19.15% (9/47)	31.25% (5/16)	12.90% (4/31)	31.25% > 12.90%, p = 0.26 (CY)
P-CDI: Normal Stress	55.32% (26/47)	50.00% (8/16)	58.06% (18/31)	50.00% < 58.06%, *p* = 0.83 (CY)
P-CDI: High stress	25.53% (12/47)	18.75% (3/16)	29.03% (9/31)	18.75% < 29.03%, *p* = 0.68 (CY)
DC: Suspicious Stress	6.38% (3/47)	6.25% (1/16)	6.45% (2/31)	6.25% < 6.45%, *p* = 0.55 (CY)
DC: Normal Stress	70.21% (33/47)	68.75% (11/16)	70.97% (22/31)	68.75% < 70.97%, *p* = 0.86 (CY)
DC: High stress	23.41% (11/47)	25.00% (4/16)	22.58% (7/31)	25.00% > 22.58%, *p* = 0.86 (CY)
DEF: Suspicious Stress	4.26% (2/47)	0.00% (0/16)	6.45% (2/31)	0.00% < 6.45%, p = 0.78 (CY)
DEF: Normal Stress	70.21% (33/47)	75.00% (12/16)	67.74% (21/31)	75.00% > 67.74%, *p* = 0.86 (CY)
DEF: High stress	25.53% (12/47)	25.00% (4/16)	25.81% (8/31)	25.00% < 25.81%, *p* = 0.77 (CY)
Total Stress: Suspicious Stress	2.13% (1/47)	0.00% (0/16)	3.23% (1/31)	0.00% < 3.23%, *p* = 0.73 (CY)
Total Stress: Normal Stress	72.34% (34/47)	75.00% (12/16)	70.97% (22/31)	75.00% > 70.97%, *p* = 0.96 (CY)
Total Stress: High Stress	25.53% (12/47)	25.00% (4/16)	25.81% (8/31)	25.00% < 25.81%, *p* = 0.77 (CY)

* = significant test; CY = χ^2^ test with Yates correction; *StD* = Standard deviation, *T* = t-Student test, *PD* = Parental Distress, *P-CDI* = Parental – Children Distress Interaction, *DC* = Difficulty Children, *DEF* = Defensive Answer; Total stress = (PD + P-CDI + DC);

The Mother’s group was composed of 31 female individuals aged between 21 and 53 years (mean 37.71 ± 8.17, of 8.17 years), while the Father’s group included 16 individuals aged between 28 and 55 years, (mean of 41.13 years ±7). There were no statistical differences between the two groups. Similarly, there was no significant difference (*p* = 0.209) among the sub-categories with normal stress: PD (74.47% = 35/47), P-CDI (55.32% = 26/47), DC (70.21% =33/47) and DEF (70.21% = 33/47).. Overall the majority of the population reported normal stress. However 1 out of 4 parents reported high stress with no major difference between mothers and fathers. The evaluation on variables revealed no significant differences between mothers and fathers in terms of high stress. These results are similar in each of the following sub-categories: PD (12.5% =2/16 < 32.26% =10/31, *p* = 0.263), P-CDI (50% = 8/16 > 41.94% = 13/31, *p* = 0.828), DC (31.25% = 5/16 > 29.03% = 9/31, *p* = 0.858) and DEF (25% = 4/16 < 32.26% = 10/31, *p* = 0.858).

In addition for high stress, there were not any remarkable differences among PD (21.28%), P-CDI (25.53%) and DC (23.41%) subscales. A similar result was reported for normal stress (74.47, 55.32 and 70.21%, respectively), while for suspicious stress (4.26, 19.15 and 6.38% respectively) there was a significant difference (*p* = 0.0033). Finally, also the cases with high stress including suspicious stress (25.53,44.68 and 29.79% respectively) bore no significant differences (*p* = 0.12). In short, PD, P-PCI and DC subscales had similar association pattern.

Table 3 shows the characteristics of parents and their children, considering parents with elevated or suspicious stress only.

**Table 3:** Characteristics of both parents and children, considering parents with elevated or suspicious stress only

Parent’s characteristics with Clinically elevated or suspicious stress (nr. =13)	Percentage/ mean ± StD	Statistical analysis
*Age*	39.77 y.o. ± 5.25 y.o.	
*Gender*		
Male	30.77% (4/13)	30.77% < 69.23%, *p* = 0.0027 ^a^ (B)
Female	69.23% (9/13)	
*Civil status*		
Not married	15.38% (2/13)	15.38% < 84.62%, *p* < 0.0001^a^ (B)
Married	84.62% (11/13)	
*Job*		
Worker with income	30.77% (4/13)	30.77% < 69.23%, *p* = 0.0027^a^ (B)
Unemployed	69.23% (9/13)	
*Education level*		*p* = 0.0026 ^a^ (C)
Elementary school	0.0% (0/13)	
Middle school	61.54% (8/13)	Middle school ^b^, *p* = 0.0084 (Z)
High school	15.38% (2/13)	
Higher Education	23.08% (3/13)	
Children’s characteristics	Percentage/ mean ± StD	Statistical analysis
*Age*	9.38 y.o. ± 4.01 y.o.	
*Gender*		
Male	53.85% (7/13)	53.85% > 46.15%, *p* = 0.58 (B)
Female	46.15% (6/13)	
*Age at diagnosis*	1.77 y.o ± 1.48 y.o.	
*Number of siblings*		*p* = 0.0173 ^a^ (C)
Zero	7.69% (1/13)	
One	53.85% (7/13)	One ^b^, *p* = 0.0375 (Z)
Two	30.77% (4/13)	
Three	7.69% (1/13)	
*Birth order*		*p* = 0.109 (C)
First	7.69% (1/13)	
Second	46.15% (6/13)	
Third	30.77% (4/13)	
Fourth	15.38% (2/13)	
*Clinical Severity*		*p* = 0.236 (C)
Low	15.38%(2/13)	
Moderate	46.15%(6/13)	
Severe	30.77%(4/13)	
No defined	7.69%(1/13)	

+ stress expressed i percentile and evaluated according to the PSI-SF scale, ^a^ = significant test, ^b^ = most frequent, ^c^ = less frequent, C = Multiple comparison χ^2^ test, *Z* = Z-test, *B* = exact Binomial test, *StD* = Standard deviation, *PD* = Parental Distress, *P-CDI* = Parental – Children Distress Interaction, *DC* = Difficulty Children; Total stress = (PD + P-CDI + DC)

In particular, only 13 in 47 parental questionnaires revealed increased stress. One of these questionnaires fell in the defensive response category with an exceptionally low stress score (PSI-SF score = 40). In this case, the individual was a married female of 37 with no work income and a high school diploma, and a male child of 2 years with a severe disease.

Table 3 shows a significant presence of female (69.23%, *p* = 0.0027), married subjects (84.62%, *p* < 0.0001), patents unemployed (69.23%, p = 0.0027) and individuals with a *middle school education level* (61.54%, *p* = 0.0084). Among the children’s characteristics, the most frequent feature was ill children with one sibling (53.85%, *p* = 0.0375).

Table 4 reports two groups: a group of parents with normal stress (PNS) (32.29% males and 64.71% females and an age range of 21–51 years, a mean of 38.53 and a standard deviation of 8.82 years), and a second group of parents with suspicious or elevated stress (PES) (30.67% males and 69.23% females, an age range of 28–58 years, a mean of 39.77 and a standard deviation 5.25 of years).

**Table 4:** Characterization and Comparison between normal stress and suspicious or elevated stress group

Parent’s characteristics	Group with normal stress	Group with suspicious or elevated stress	Normal vs. suspicious or elevated stress Group
*Age* (mean ± StD y.o.)	38.53 ± 8.82	39.77 ± 5.25	38.53 < 39.77, *p* = 0.64 (T)
*Gender*			
Male	35.29% (12/34)	30.77% (4/13)	35.29% > 30.77%, p = 0.96 (CY)
Female	64.71% (22/34)	69.23% (9/13)	64.71% < 69.23%, *p* = 0.96 (CY)
*Civil status*			
Not married	8.82% (3/34)	15.38% (2/13)	8.82% < 15.38%, *p* = 0.90 (CY)
Married	91.18% (31/34)	84.62% (11/13)	91.18% < 84.62%, p = 0.90 (CY)
*Job*			
Worker with income	50.00% (17/34)	30.77% (4/13)	50% > 30.77%, p = 0.90 (CY)
Unemployed	50.00% (17/34)	69.23% (9/13)	50% < 69.23%, p = 0.90 (CY)
*Education level*			
Elementary school	2.94% (1/34)	0.00% (0/13)	2.94% > 0.00%, p = 0.61 (CY)
Middle school	50.00%(17/34)	61.54% (8/13)	50.00% < 61.54%, *p* = 0.70 (CY)
High school	32.35% (11/34)	15.38% (2/13)	32.35% > 15.38%, *p* = 0.42 (CY)
Higher Education	14.71% (5/34)	23.08% (3/13)	14.71% < 23.08%, *p* = 0.80 (CY)
Children’s characteristics	Group with normal stress	Group with suspicious or elevated stress	Normal vs. suspicious or elevated stress Group
*Age* (mean ± StD y.o.)	7.82 ± 5.04	9.38 ± 4.01	7.82 < 9.38, *p* = 0.32 (T)
*Gender*			
Male	55.88% (19/34)	53.85% (7/13)	55.88% > 53.85%, *p* = 0.84 (CY)
Female	44.12% (15/34)	46.15% (6/13)	44.12% > 46.15%, p = 0.84 (CY)
*Age at diagnosis* (mean ± StD y.o.)	1.59 ± 1.35	1.77 ± 1.48	1.59 < 1.77, *p* = 0.69 (T)
*Number of siblings*			
None	52.94% (18/34)	7.69% (1/13)	52.94% > 7.69%, p = 0.013 ^a^ (CY)
One	41.18% (14/34)	46.15% (6/13)	41.18% < 46.15%, p = 0.96 (CY)
Two	5.88% (2/34)	30.77% (4/13)	5.88% < 30.77%, *p* = 0.072 (CY)
Three	0.00% (0/34)	15.38% (2/13)	0.00% < 15.38%, *p* = 0.13 (CY)
*Birth order*			
First	61.76% (21/34)	53.85% (7/13)	61.76% > 53.85%, *p* = 0.87 (CY)
Second	38.24% (13/34)	30.77% (4/13)	38.24% > 30.77%, p = 0.87 (CY)
Third	0.00% (0/34)	7.69% (1/13)	0.00% < 7.69%, p = 0.61 (CY)
Fourth	0.00% (0/34)	7.69% (1/13)	0.00% < 7.69%, *p* = 0.61 (CY)
*Clinical Severity*			
Low	67.65%(23/34)	15.38%(2/13)	67.65% > 15.38%, *p* = 0.0039 ^a^ (CY)
Moderate	29.41%(10/34)	46.15%(6/13)	29.41% < 46.15%, *p* = 0.46 (CY)
Severe	2.94%(1/34)	30.77%(4/13)	2.94% < 30.77%, p = 0.0252 ^a^ (CY)

^a^ = significant test; CY = χ^2^ test with Yates correction, *StD* = Standard deviation, *T* = t-Student test;

A significant presence of children with one sibling in the PNS group was observed compared to the PES group with 52.94% > 7.69% (*p* = 0.013). In addition, a significant presence of children with low *Clinical Severity* was revealed in the PNS group, while a remarkable number of children with a severe disease was observed in the PES group compared to the PNS group with 30.77% > 2.94%, (*p* = 0.0252).

Finally, Table 5 shows both univariate and a multivariate linear regression analysis between *Total stress* evaluated with the standardized questionnaire PSI-SF, and the independence variables associated to parents such as *Age*, *Gender parents*, *Civil Status*, *Job*, *Education level*, and between *Total stress* and the independent variables associated to children such as *Children’s Age*, *Age at diagnosis*, *Number of siblings*, *Birth order* and *Clinical Severity*.

**Table 5:** Univariate and multivariate linear regression analysis between Total stress and *Age parents, Gender parents, Civil status*, *Job*, *Education level, Age children, Gender children, Age at diagnosis*, Number of siblings, Birth order, Clinical Severity

Linear correlation analysis	Univariate analysis R (*p*-value)	Multivariate analysis Rpartial; *p*-value
		Multiple linear correlation coefficient = 0.22
Total Stress / *Age parents*	0.09 (0.56)	*Rpartial* = 0.12; *p*-value = 0.43
Total Stress / *Gender parents*	0.02 (0.87)	*Rpartial* = 0.09; *p*-value = 0.59
Total Stress / *Civil status*	−0.12 (0.42)	*Rpartial* = −0.15; *p*-value = 0.34
Total Stress / *Job*	− 0.08 (0.58)	*Rpartial* = − 0.13; *p*-value = 0.39
Total Stress / *Education level*	−0.03 (0.83)	*Rpartial* = − 0.07; p-value = 0.67
		Multiple linear correlation coefficient = 0.51
Total Stress / *Age children*	0.25 (0.08)	*Rpartial* = − 0.03; *p*-value = 0.83
Total Stress / *Gender children*	−0.1 (0.51)	*Rpartial* = − 0.15; *p*-value = 0.36
Total Stress / *Age at diagnosis*	0.1 (0.52)	*Rpartial* = 0.05; *p*-value = 0.76
Total Stress / *Number of siblings*	0.28 (0.05)	*Rpartial* = 0.34; *p*-value = 0.0282 ^a^
Total Stress / *Birth order*	−0.15 (0.31)	*Rpartial* = − 0.37; *p*-value = 0.0177 ^a^
Total Stress / *Clinical Severity*	0.30 (0.0424) *	*Rpartial* = 0.14; *p*-value = 0.38

^a^ = significant test; *R* = Pearson’s linear correlation coefficient; *R_partial* = the partial correlation coefficient is the

coefficient of correlation of the variable with the dependent variable, adjustment due to the impact of the other variables on the mode; *PD* = Parental Distress, *P-CDI* = Parental – Children Distress Interaction, *DC* = Difficulty Children, Total stress = (PD + P-CDI + DC);

The univariate analysis pointed out a significant positive association between *Total stress* and children’s *Clinical Severity*, indicating that parent stress was directly proportional to child *Clinical Severity*. In the multivariate analysis, i.e. considering simultaneously for children: *age, gender, age at diagnosis, clinical severity, number of siblings and birth order*, it resulted that the *Number of siblings* was a negative predictor of *Total stress* variable, while *Birth order* was a significant positive predictor of *Total stress* variable; in other words, an increased number of siblings represents a predictor of a decrease of parenting stress level, vice versa for *Birth order*.

Finally we observed in *Total stress* variable a significant presence of parents with normal stress (72.34% = 34/47) in contrast to parents with high stress (25.53% = 12/47) including subject with suspicious stress (2.13% = 1/47), i.e. it resulted: 72.34% (34/47) > 27.66% (13/47), *p* < 0.0001.

## Discussion

In this study we analyzed parenting stress of children affected by Cystic Fibrosis through a biopsychosocial approach. The latter, which is supported both by the World Health Organization (WHO) and many data [13, 14], indicates that healthcare professionals should provide adequate healthcare assistance to children with the disease as well as a psychological support to their parents in order to improve their overall quality of life. The care should include biomedical and psycho-social interventions to achieve a positive impact on patient’s life.

Contrary to our expectations [15–18], the study revealed a significant presence of parents with normal stress level. We hypothesize that this result may be attributed to the low *Clinical Severity* of the children enrolled in this study (Tables 4 and 5). In addition, we found a positive correlation between *Clinical severity* and *Total stress,* i.e. it was observed an increasing of abnormal stress in parents connected to an increasing of clinical severity in children. This result was not detected in a previous review [4]. We also investigated on additional parameters to identify possible correlations with parenting stress such as education levels, civil status, number of siblings, clinical status, etc. A significant correlation was found between stress levels and number of siblings in a multivariate analysis, i.e. the presence of more children in the same family is associated with high parental stress levels. This may be due to the higher commitment of parents in having to manage more children, when repeated hospitalizations cause more stress [19]. In terms of *Birth order*, the study has shown that parents of children with chronic illness bear higher stress level compared to other parents, and in particular, a high *Birth order* in a family with a child with the disease, implies parents with high or suspicious stress, whereas a low *Birth order* in a family predicts parents with normal stress levels. It has been recently shown that the mother first born interaction differs from the mother second born interaction in healthy children [20].

Our results may suggest that the increased distress observed in parents of children with chronic disease is probably affected by the great expectations that parents have on their first child.

In the event of a sick, the parents need a mental re-adjustment between their expectations of an ideal child and the real child status. The *Clinical Severity* analysis revealed a correlation between parental stress levels and degree of clinical severity, that is parental stress is higher in parents with a child with a severe disease and less in parents with a child with low *Clinical Severity*.

With regard to the total scores among stress sub-scales (Parenting Distress – PD, Parent-Child Dysfunctional Interaction P-CDI, Difficult Child (DC), the lowest score of parents with normal stress value was reported in the P-CDI sub-scale. P-CDI sub-scale measures parents’ expectations and interactions with their child. High scores in P-CDI may indicate a parent’s feelings of disappointment either caused by the child or by a lack of proper bonding with their child [21]. The set of questions made it possible to investigate the parent-child relationship and to point out whether the parent perceived the relationship with their child to be different from the one they expected in a no sick child, (i.e. “My child is not able to do as much as I expected”, “When I do things for my child, I feel that my efforts are not much appreciated”, “My son does not seem to learn as fast as most children do!”). It is clear that the illness event in these parents also negatively influences parent-child dynamics [22].

Similarly, none of the sub-categories has shown any remarkable difference between fathers and mothers in terms of high stress (including stress to be assessed and high stress) 25.0 < 29.03%. In this case, a higher level of stress in mothers would have been more likely expected as they are normally more involved in the history of the disease. As they are nearly always hospitalized together with the child, these mothers could be overloaded with more stress [18, 23].

However, despite the small number of parents with clinically elevated or suspicious stress (27.66%), their features were nonetheless analyzed.

Considering parents with high or suspicious stress levels only (Table 3), a higher presence of mothers was observed as opposed to fathers (69.23% > 30.77), the mean age was of about 40 years, married individuals were the majority (84.62%), and there was a substantial presence of individuals without regular paid job (69.23%) and a middle school education level (61.54%). Finally, among the examined parents, high stress was detected in parents having a second child (53.85%) with Cystic Fibrosis, i.e. an ill child with one sibling.

## Conclusion

This study has shown that, despite the fact that a child’s chronic disease inevitably puts a heavy stress on a family, making them vulnerable and emotionally exposed, an effective multidisciplinary care plan, that takes into account the emotional and sympathetic aspect of caregivers, would help parents development of internal resources to allow them to cope with stress. Such comprehensive model of management with a regularly provided psychological support appears to be a key factor for coping and for achieving a strong therapeutic alliance between healthcare professionals and patients, in other words a strong psychological support fits particularly well in the biopsychosocial model of comprehensive are in this progressively worsening disease.

### Limitations

The current study has some limitations. As the size of the sample drawn for the current analysis was relatively small compared with other studies examining the psychometric properties of the PSI-SF, the aim of the study may be limited in its. For this reason, the data presented should be interpreted as preliminary results, although they open the way to encourage future research on the psychometric properties of the PSI-SF with larger samples and similar demographic compositions within a multi-center study. However, despite such limitations, the study has demonstrated that the clinical management of CF children should include a psychological support to parents since parenting stress does play a fundamental role in the effectiveness and efficacy of treatments as well as in patients’ quality of life.

## Data Availability

The datasets during and/or analyzed during the current study available from the corresponding author on reasonable request.

## Abbreviations

CF: Cystic fibrosis
PSI-SF: Parenting stress index – short form questionnaire
CFTR: .
WHO: World health organization
SA: Strongly agree
NS: Not sure
D: Disagree
SD: Strongly disagree
PD: Parenting distress
P-CDI: Parent-child dysfunctional interaction
DC: Difficult child
DEF: Defensive response
PNS: Group of parents with normal stress
PES: Group of parents with suspicious or elevated stress
R: Pearson’s linear correlation coefficient
p: *P*-value.
